# Conradi-Hünerman-Happle Syndrome and Obsessive–Compulsive Disorder: a clinical case report

**DOI:** 10.1186/s12888-023-04579-1

**Published:** 2023-02-06

**Authors:** Sabrina de Jesus, Ana Lúcia R. Costa, Mónica Almeida, Paula Garrido, João Alcafache

**Affiliations:** Departament of Psychiatry and Mental Health, Baixo Vouga Hospital Center, Aveiro, Portugal

**Keywords:** Chondrodysplasia punctata, Obsessive compulsive disorder, Comorbidity, Etiology, Rare disease

## Abstract

**Background:**

Obsessive–Compulsive Disorder (OCD) is a common and chronic psychiatric disorder with significant morbidity characterized by intrusive, uncontrollable and reoccurring thoughts (i.e., obsessions) and/or ritualistic behaviours (i.e., compulsions). Conradi-Hünerman-Happle Syndrome (CHHS) is a rare inherited X-linked dominant variant of chondrodysplasia punctata, a heterogeneous group of rare bone dysplasias characterized by punctate epiphyseal calcifications of complex etiology and pathophysiology that remain to be defined. Available literature reveals a lacuna in regards to the coexistence of the entities with no clinical reports described.

**Case presentation:**

A 12 year old female patient with diagnosis of CHHS, presents to psychiatric consultation due to aggravation of her OCD clinical picture, with aggravation of hand-washing frequency during the Covid-19 pandemic with significant functional impact. Psychopharmacological treatment aimed at OCD with Selective Serotonin Reuptake Inhibitor (SSRI) and antipsychotic was instituted with favourable, albeit partial response.

**Conclusions:**

The authors aim to describe a clinical case in which the patient presents with Conradi-Hünerman-Happle Syndrome and Obsessive–Compulsive Disorder. Clinical descriptions of CHHS and OCD are not available in the literature. Through this case description the authors aim to present a rare case as well as discuss an eventual association between etiology and/or pathophysiology of the two disorders.

## Background

Obsessive–Compulsive Disorder (OCD) is a common and chronic psychiatric disorder with significant morbidity characterized by obsessions which are intrusive, uncontrollable and reoccurring thoughts and/or compulsions which are ritualistic behaviours performed as a means to decrease distress frequently associated with obsessions [1, 2]. OCD is the fourth most common mental disorder after depression, alcohol/substance misuse, and social phobia [3] affecting approximately 1–3% of the population [4]. There is a significant gender difference in the OCD age of onset with males developing symptoms before the age of 10 in contrast to females who usually develop symptoms after this [5].

OCD is a relevant example of a psychiatric disorder in which research has contributed to better recognition, assessment and outcomes. Several neurobiological mechanisms underlying OCD have been identified, including specific brain circuits, cellular and molecular dysfunction and genetic markers that underpin OCD psychopathological expression [6].

Frequently comorbid with other psychiatric disorders, the literature also demonstrates an association between OCD or OC symptoms and coexisting medical conditions, spanning across various systems including pulmonary, autoimmune, rheumatologic, neurologic, cardiovascular, gastrointestinal and thyroid conditions [7, 8] with significant impact on illness duration and severity of symptoms [9]. Although this association has been described in the literature, it is worth mentioning that the literature regarding somatic health of those with OCD is somewhat limited [8] thus cementing the importance of discussing comorbid conditions, such as CHHS.

Effective treatments for OCD typically include the use of high dose selective serotonin reuptake inhibitors (SSRIs), cognitive–behavioural therapy (CBT), with neuromodulation techniques and neurosurgery usually reserved for those with intractable symptoms [6].

First described by Conradi in 1914 and fully delineated by Happle in the late 1970s, Conradi-Hünermann-Happle Syndrome (X-linked dominant chondrodysplasia punctata, CDPX2, OMIM 302,960) [10] is a rare inherited X-linked dominant variant of chondrodysplasia punctata, a heterogeneous group of rare bone dysplasias [11, 12]. It is a multisystem disorder characterized by skeletal, ocular and cutaneous anomalies with asymmetric involvement of the body [10]. This specific syndrome occurs almost exclusively in females, approximately 95%, because the underlying X-linked gene defect is typically lethal in males [13]. Life expectancy in mild cases appears to be normal, assuming that heart and lung function is not impaired by severe scoliosis [12]. The intelligence of individuals with Conradi-Hünermann syndrome is usually not affected [13].

This syndrome is of heterogeneous presentation ranging from fatal to milder expressions where few physical anomalies are detected. Characteristic features of CHHS may include growth deficiency; distinctive craniofacial appearance (flattened nasal bridge and frontal bossing); chondrodysplasia punctata (stippling of the epiphyses of the long bones, vertebrae, trachea, and distal ends of the ribs); asymmetric limb and vertebral malformations; linear or blotchy scaling ichthyosis; skin lesions arranged in a linear or whorled atrophic pattern involving hair follicles; coarse hair with scarring alopecia; flattened or split nails; and cataracts [12–14].

The etiology and pathogenesis are complex and not completely defined, with few cases and studies described in the literature. The majority of cases appear to originate from sporadic mutations on the EBP gene, although other cases in the literature appear to adhere to the X-linked dominant pattern of inheritance [14].

Treatment of CHHS is mainly symptomatic, individualized and include family support. Management by various specialties may be warranted depending on the phenotype, including orthopedic management for inferior limb asymmetries and kyphoscoliosis; dermatologic management of skin alterations; and pulmonary management if respiratory complications arise [13].

## Clinical case

The clinical case describes a 12 year old female patient, diagnosed previously with Conradi-Hünermann-Happle syndrome through clinical presentation and confirmed via genetic testing, who was referred to Psychiatry consultation due to aggravation of her psychopathological condition, Obsessive–Compulsive Disorder, diagnosed clinically in keeping with the established criteria defined by the Diagnostic and Statistical Manual of Mental Disorder, fifth edition [15]. The patient’s condition was marked by repetitive behaviours that had progressively begun to have an impact on her functioning, coincidentally with the beginning of the Covid-19 pandemic.

She slowly started by repeating certain sequences of words, with the short phrase "do not think" being the most frequent and currently prevalent. Posteriorly, thoughts about uncomfortable topics, usually related to tragedies and illnesses involving her family members developed. According to the patient, if she repeated the words "do not think" a certain number of times, nothing negative would happen to her family thus attenuating the anxiety and discomfort associated with the intrusive thought. The number of times that the patient would have to repeat these words has varied over time, remaining around seven times most of the time, and at the moment of consultation has been variable. She is unable to explain the criteria used to choose the number of times necessary to expel negative outcomes. Further, the patient adds that although she recognizes these thoughts as hers, they are egodystonic in character, are intrusive, recurring and cause marked discomfort. She repeats the words in order to alleviate this discomfort, recognizing the lack of rationality between the association of these words and the probability of something tragic happening.

Along with these ideas, she says that she also feels the need to wash her hands often, especially after the Covid-19 pandemic outbreak, for fear of becoming infected and getting sick but verbalizes significant fear of contaminating someone. She avoids touching certain objects, and when she does she subsequently washes her hands repeatedly until she feels that she is decontaminated. At observation the patient presents hands with thin, shiny and erythematous skin, due to apparent repeated contact with detergents and friction. She claims to try to resist these behaviours, but to no avail.

Also noteworthy, is the presence of a simple motor tic, in the form of a blink, documented since around 4 years of age with a pattern of varying intensity over the years.

The Obsessive–Compulsive (OC) rituals have had an impact on the patient´s quality of life and time spent on leisure, but has yet to influence her school performance evidenced by having received a school merit award (honour roll) and having obtained an A + (maximum grade) on all subjects. Due to this academic performance, and due to the literature not reporting impact of Conradi-Hünermann-Happle syndrome on intelligence and cognitive performance, formal testing was not carried out.

In this context and due to the marked distress and anxiety caused in the patient, it was decided to start psychopharmacological treatment with SSRI, Sertraline 100 mg, in a titrated dose. Symptom severity and clinical impact of Sertraline introduction was evaluated retrospectively, taking into consideration previous clinical presentation as well as patient report. It was determined to have resulted in a positive but partial response in attaining symptomatic control with benefits in terms of symptom intensity and frequency translated through diminished ritualized hand-washing. The introduction of Pimozide 1 mg id in a potentiation strategy was considered, with the prospect of providing accessory control of motor tics, but ultimately not utilized.

In addition to the clinical picture previously described, she also presents low nasal bridge, flat face, down-slanting space between eyelids, abnormal redness of the skin, flaky skin, cataracts and severe lumbar kyphosis with generalized joint pain, which led to the prohibition of sports activities following Orthopedic indications. These changes are explained by the Conradi-Hünermann-Happle syndrome. The patient is unaware of other medical conditions.

As relevant family history, a maternal aunt with a stable diagnosis of Obsessive–Compulsive Disorder is noteworthy.

The patient was followed-up regularly in consultation, with methodical evaluation of the patient´s clinical state conducted by a Psychiatrist responsible for the OCD ambulatory regime, complying with a periodicity which varied between 60 to 90 days, depending on the clinical picture. On follow-up, the patient response to the instituted psychopharmacological treatment, was overall positive and did not differ from the projected pattern of response to typical clinical presentations of OCD. Few symptoms maintained however, namely that of obsessions, albeit of diminished intensity, and sporadic motor tics which remitted after introduction of low dose Risperidone (1 mg). The patient maintains positive global functioning and remission of OC symptoms and motor tics.

## Discussion

The co-occurrence of physical medical disorders and major psychiatric disorders is extensively described in the literature [7, 9]. This frequent concomitancy between physical ailments and mental disorders can be explained namely by: the impact of psychopharmacological treatments, illness-related factors such as unhealthy lifestyles associated with the psychiatric disorder and the probable effect of inherited genetic factors [9, 16].

Among patients with OCD, studies are scarce, however, various medical comorbidities were found to be more prevalent than in the general population, including nutritional and metabolic diseases, cardiovascular diseases, viral diseases, gastrointestinal problems, migraine, respiratory tract diseases and musculoskeletal diseases [7, 9, 16, 17]. This becomes relevant as the presence of any chronic physical condition has been shown in one study to increase the prevalence of OC symptoms, [18] an important factor in quality of life and treatment options. Another study demonstrated that of the anxiety disorders, OCD was found to be the disorder with the highest estimate of the number of life years lost due to the disease in men and second highest in women [19] as well as being associated with an elevated duration of untreated illness when associated with a medical comorbidity [9].

Therefore, the presence of OCD and a general medical condition, such as CHHS, becomes relevant due to increased probability of worsened quality of life and functioning, longer duration of untreated illness, increased utilization of healthcare resources and subsequent elevated risk of hospitalization and mortality.

In addition to this, this case allows for the tentative discussion of possible shared etiological pathways which necessitate further inquiry and study. It should be born in mind, that discussion of potential contributors to the pathogenesis of both these disorders is hindered by the lack of literature in what concerns CHHS pathogenesis, due to the rarity of the syndrome. On the other hand, various mechanisms appear to contribute to the pathogenesis of OCD, with the literature expanding on this front. Taking into consideration what has been described in regards to the etiology of both conditions, a proposed shared common pathway between CHHS and OCD may be via cholesterol metabolism.

Sterols are important constituents of the cell membranes of most eukaryotic cells with the cholesterol acting as the major sterol in human cells [20]. Cholesterol is found in external cellular membranes (plasma membranes) and in the layers that comprise myelin sheaths in the central and peripheral nervous systems, [20] thus playing an important role in neural signalling. Taking into consideration the widespread presence of this sterol, it is unsurprising to find that disorders of cholesterol biosynthesis and serum levels have been associated with a myriad of clinical syndromes, including neuropsychiatric ones.

CHHS results from a mutation in the emopamil-binding protein (EBP) gene and subsequent defective cholesterol biosynthesis [21] leading to the accumulation of cholesterol precursors such as 8-dehydrocholesterol and 8(9)-cholestenol in the plasma, [22, 23] which serves as a diagnostic marker. The clinical phenotype in CHHS results directly from impairment in cholesterol biosynthesis, and indirectly from abnormalities in the hedgehog signalling protein pathways [24].

Studies have begun to demonstrate a potential relationship between mental health disorders and cholesterol [25] with elevated cholesterol levels being reported in schizophrenia, OCD, panic disorder, generalized anxiety disorder, and post-traumatic stress disorder [26, 27]. Postulated explanations for this appear to stem from alterations of the Hypothalamic–Pituitary–Adrenal axis, increased cortisol production and catecholamine synthesis [27], however, further studies are needed in order to confirm these preliminary findings. The literature remains scarce in regards to altered cholesterol biosynthesis and its role in OCD, with preliminary results demonstrating altered serum levels in a variety of psychiatric disorders, thus meriting further study. Whether or not a shared pathway in terms of cholesterol biosynthesis exists between the two disorders, remains to be explored.

Whether or not other pathways such as those involving neuroinflammation or immune factors which have been explored as potentially contributing to the pathogenesis of OCD also play a role on the etiology of CHHS is yet to be described. Taking into consideration the genetic basis for the development of CHHS whether by X-linked dominant inheritance or by de novo mutations on the EBP gene [13], a genetic overlap between the two conditions may exist given the alterations in cholesterol metabolism documented in both, however, this remains to be determined through further studies exploring underlying contributing factors.

## Conclusion

The low incidence of Conradi-Hünermann-Happle syndrome makes the study of psychiatric symptoms eventually associated with it particularly difficult. In the literary research carried out, no association was found (or described) between this syndrome and Obsessive–Compulsive Disorder, so the great dilemma of the case presented is to understand if the Obsessive–Compulsive symptoms are an episodic finding or are part of a more complex global clinical picture, still poorly studied.

The sharing of clinical cases like the one described can contribute to a better understanding of the syndrome and its clinical presentation, which is still poorly defined from a psychiatric and neurological point of view. The patient's family history may suggest a family pattern of OCD, regardless of the presence of Conradi-Hünermann-Happle syndrome. Continued effort into elucidating the probable multifactorial pathogenesis of both conditions may further contribute to the identification of overlapping risk factors, such as a combination of genetic factors or other possible causes in developments such as neuroinflammation [7, 9, 13, 17].

In regards to limitations, this case report demonstrates those inherit to this form in which the general applicability of the associations or eventual cause and effects postulated remain as hypothesis difficult to confirm due to the lack of similar cases available in the literature. However, this case highlights the importance of taking comorbidity between psychiatric disorders and other medical conditions into consideration as this co-existence might condition prevention and treatments efforts as well as the prognosis of either pathology. For a better understanding of this complex clinical picture, it will be essential to study the evolution of symptoms, as well as to share and discuss similar clinical cases as well as explore potentially shared pathophysiological pathways.

## Data Availability

Not applicable.

## Abbreviations

CBT: Cognitive-Behavioural Therapy
CHHS: Conradi-Hünerman-Happle Syndrome
EBP: Emopamil-Binding Protein
OCD: Obsessive–Compulsive Disorder
OC: Obsessive–Compulsive
SSRIs: Selective Serotonin Reuptake Inhibitors
